# Federated Multi-Label Learning (FMLL): Innovative Method for Classification Tasks in Animal Science

**DOI:** 10.3390/ani14142021

**Published:** 2024-07-09

**Authors:** Bita Ghasemkhani, Ozlem Varliklar, Yunus Dogan, Semih Utku, Kokten Ulas Birant, Derya Birant

**Affiliations:** 1 Graduate School of Natural and Applied Sciences, Dokuz Eylul University, Izmir 35390, Turkey; bita.ghasemkhani@ogr.deu.edu.tr; 2 Department of Computer Engineering, Dokuz Eylul University, Izmir 35390, Turkey; ozlem@cs.deu.edu.tr (O.V.); yunus@cs.deu.edu.tr (Y.D.); semih@cs.deu.edu.tr (S.U.); ulas@cs.deu.edu.tr (K.U.B.); 3 Information Technologies Research and Application Center (DEBTAM), Dokuz Eylul University, Izmir 35390, Turkey

**Keywords:** animals, machine learning, multi-label learning, federate learning, Federated Multi-Label Learning, Binary Relevance, Reduced-Error Pruning Tree

## Abstract

**Simple Summary:**

This study addresses the classification task in animal science, which helps organize and analyze complex data, essential for making informed decisions. It introduces Federated Multi-Label Learning (FMLL), a novel approach combining federated learning principles with a multi-label learning technique. Using machine learning strategies, FMLL achieved significant improvements in classification accuracy metrics compared to existing methods. The experimental results on different animal datasets demonstrated the effectiveness of FMLL and its superiority in multi-label classification tasks. The findings of our study offer valuable insights into understanding and managing animal populations, which could have important implications for biodiversity conservation and ecological management.

**Abstract:**

Federated learning is a collaborative machine learning paradigm where multiple parties jointly train a predictive model while keeping their data. On the other hand, multi-label learning deals with classification tasks where instances may simultaneously belong to multiple classes. This study introduces the concept of Federated Multi-Label Learning (FMLL), combining these two important approaches. The proposed approach leverages federated learning principles to address multi-label classification tasks. Specifically, it adopts the Binary Relevance (BR) strategy to handle the multi-label nature of the data and employs the Reduced-Error Pruning Tree (REPTree) as the base classifier. The effectiveness of the FMLL method was demonstrated by experiments carried out on three diverse datasets within the context of animal science: Amphibians, Anuran-Calls-(MFCCs), and HackerEarth-Adopt-A-Buddy. The accuracy rates achieved across these animal datasets were 73.24%, 94.50%, and 86.12%, respectively. Compared to state-of-the-art methods, FMLL exhibited remarkable improvements (above 10%) in average accuracy, precision, recall, and F-score metrics.

## 1. Introduction

Animal science is an area where machine learning (ML) has proven effective in analyzing animal datasets and making predictions for future decisions. ML techniques have been utilized for different purposes such as animal health surveillance, outlier animal behavior detection, animal activity recognition, animal detection systems, and animal species classification. Moreover, multi-label learning as a subfield of ML has gained traction in animal science for handling complex scenarios where multiple labels need to be predicted simultaneously [1,2,3]. Furthermore, combining multi-label classification with federated learning (FL) enables distributed and privacy-preserving machine learning applications. Recent studies have demonstrated the effectiveness of FL in animal science initiatives, including federated frameworks for diagnosing and predicting animal diseases, monitoring animal welfare, predicting collaborative disease outbreaks, and implementing decentralized systems for animal tracking and detection [4,5,6]. These advancements highlight the potential of federated multi-label learning to revolutionize animal science by integrating robust predictive modeling with secure data-sharing mechanisms.

Federated learning is a collaborative ML approach that was introduced in 2016 [7]. In the FL framework, multiple clients work together to address machine learning problems, overseen by a central aggregator. This setup ensures that training data remains decentralized, safeguarding the privacy of each client’s data. In this framework, client data remains stored locally, and local models are trained in multiple nodes. Gaining popularity in recent years, this kind of distributed machine-learning technique builds a central model by aggregating local models, thereby reducing the computational complexity of training [8]. Consequently, federated learning proves highly beneficial in resolving privacy issues associated with data islands and holds promise for deployment across diverse edge devices [9,10].

Multi-label learning is a sophisticated machine learning paradigm that extends traditional classification techniques by allowing instances to be associated with multiple labels simultaneously. Unlike conventional single-label classification tasks where each instance is assigned to a single class, multi-label learning builds a model in which instances may exhibit multiple attributes or characteristics. This paradigm finds widespread application in domains where instances are inherently multi-faceted, such as image recognition [11], text classification [12], and biology [13]. For example, in biology classification tasks, multi-label learning can be applied to predict the functions of elements based on their multiple roles within biological pathways. The multi-label learning algorithm aims to capture the complex relationships between instances and their associated labels, finding applications across other fields e.g., animal [14], healthcare [15], social media [16], geoscience [17], transportation [18], and more, where data instances may belong to various classes at the same time. 

Multi-label learning entails its own set of challenges. One common challenge is the increased complexity of model training and evaluation processes since multi-label datasets typically exhibit larger sizes and greater complexity compared to single-label datasets. Another challenge is that the presence of multiple labels can further complicate the learning process and require specialized algorithms. To tackle these obstacles, researchers have developed a solution, namely the binary relevance (BR) approach, which streamlines the learning process and facilitates the utilization of standard binary classifiers, such as support vector machines [19]. Additionally, techniques such as label powersets and classifier chains, have been proposed to tackle different aspects of the multi-label learning problem. 

The Reduced-Error Pruning Tree (REPTree) algorithm is another method employed in machine learning, particularly in the context of decision tree-based classification tasks. REPTree aims to construct an optimal decision tree by iteratively pruning branches that do not contribute significantly to reducing classification error [20]. REPTree has applications in various domains such as animal [21], environment [22], healthcare [23], and education [24]. When considering multi-label classification tasks, REPTrees can serve as effective binary classifiers within the binary relevance framework. Each REP Tree can be trained independently to predict the absence or presence of a specific label, utilizing its pruning mechanism to optimize classification performance. They are simple yet powerful solutions, leveraging decision tree structures while handling the complexity of multiple labels per instance, to provide interpretable models that can manage both categorical and numerical data, making them suitable for a broad range of real-world problems. 

The exploration of federated learning and multi-label learning, particularly in conjunction with methodologies such as the binary relevance approach and REPTree, remains relatively uncharted territory in the literature. Thus, in response to the evolving landscape of distributed data and complex classification tasks, we propose a novel approach, Federated Multi-Label Learning (FMLL) for classification tasks in the current study. Drawing upon established methodologies, namely Binary Relevance and Reduced-Error Pruning Tree (REPTree) approaches, our method aims to combine the strengths of federated learning and multi-label concepts to address the challenges inherent in distributed environments and multi-dimensional classification problems. The primary contributions of this study, setting it apart from other classification methods, are as follows:

(i) The paper presents the first-of-its-kind Federated Multi-Label Learning (FMLL) method that combines federated learning principles with the Binary Relevance approach as a multi-label learning technique and uses the REPTree algorithm to address classification tasks where instances may belong to multiple classes simultaneously.
(ii) FMLL contributes significantly to the field of animal science by offering a novel methodology for classifying diverse animal datasets. This advancement enables more accurate and efficient classification of animals based on various attributes, aiding researchers and practitioners in better understanding and managing animal populations.
(iii) FMLL harnesses federated learning principles, allowing multiple nodes to collaboratively train a model using their own local data. This provides the distribution of computational complexity over multiple nodes to improve efficiency and ensures privacy preservation and data security, which are crucial considerations in animal science research where large sensitive data may be involved.
(iv) The proposed approach adopts the Binary Relevance (BR) strategy to effectively handle the multi-label nature of the data. By accurately classifying instances belonging to multiple classes, FMLL enhances the understanding of complex relationships and characteristics within animal species datasets.
(v) FMLL pioneers the use of the Reduced-Error Pruning Tree (REPTree) classifier within federated learning, marking the first instance in the literature. The REPTree was chosen for its effectiveness in addressing the complexities of multi-label classification tasks. This approach enhances both the accuracy and interpretability of classification results, representing a significant advancement in machine learning techniques applied to animal science.
(vi) The effectiveness of FMLL is empirically validated through experiments conducted on three diverse datasets within the domain of animal science: Amphibians, Anuran-Calls-(MFCCs), and HackerEarth-Adopt-A-Buddy. These experiments demonstrated the applicability and efficacy of FMLL in real-world scenarios, showcasing significant improvements in classification accuracy.
(vii) FMLL achieved remarkable improvements in classification accuracy across various animal datasets when compared to existing state-of-the-art methods. For instance, on the Amphibians dataset, FMLL achieved an average accuracy improvement of 10.92%. This improvement highlights the practical relevance and superiority of FMLL in multi-label classification tasks within the domain of animal science.

The structure of this paper unfolds as follows: Section 2 provides a concise review of related works, followed by Section 3, where we detail the materials and methods employed. Section 4 presents the experimental studies conducted, while Section 5 discusses the obtained results. Section 6 elucidates the conclusions drawn from our findings and delineates potential directions for future research on the proposed method.

## 2. Related Works

Lately, a plethora of researchers have committed their endeavors to developing federated learning (FL) techniques, aiming to bolster the efficacy of machine learning (ML) models. FL has found applications across different domains including health [25,26,27,28], agriculture [29,30,31,32], security [33,34,35,36], environment [37,38], animals [39,40,41], industries [42,43,44], transportation [45,46,47], and education [48,49,50,51]. For example, in the domain of health [28], a federated learning approach was introduced for the client end of health service providers. Their method incorporates modified artificial bee colony optimization and support vector machine techniques to enhance the accuracy of cardiovascular disease classification. In agriculture [31], a federated learning-based entropy model was presented to assess food safety by quantifying risk levels associated with pesticide residues in agricultural products. In security [36], the integration of homomorphic encryption into the privacy-preserving federated learning algorithm was implemented to empower centralized servers to securely aggregate encrypted local model parameters. In the environmental domain [40], a novel federated learning framework for animal activity recognition (FedAAR) was proposed to address the challenges of sensor-based animal monitoring systems through decentralized data from several farms.

Table 1 presents an overview of federated learning frameworks [30,52,53,54,55,56,57,58,59,60], offering insights to better understand the contributions made in this field. Various machine learning methods have been employed in previous studies, including the sparrow search algorithm (SSA) [52], the differential privacy Laplace mechanism (DPLA) [52], the amendable multi-function sensor control method (AMFSC) [54], and the multiscale residual attention network (MSRAN) [55]. While most studies [57,58,59,60] evaluated the results using the accuracy metric, some of them [30,53,55,56] also utilized F-score, precision, recall metrics, and others [52,53,56] used different indicators like the confusion matrix, false positive rate (FPR), mean absolute error (MAE), root mean square error (RMSE), and coefficient of determination (R-squared). 

Federated learning has been applied successfully across a broad spectrum of machine learning algorithms, including decision trees (DTs) [61], artificial neural networks (ANNs) [62], support vector machines (SVMs) [63], logistic regression (LR) [64], random forests (RFs) [65], and k-nearest neighbors (KNNs) [66]. These implementations have demonstrated the versatility and flexibility of federated learning techniques in diverse settings. Furthermore, different types of multi-based methodologies have emerged within the realm of federated learning, each aiming to address specific requirements and challenges. These similar methodologies to our FMLL method include multi-dimensional federated learning [67], multi-objective federated learning [68], multi-modal federated learning [69], multi-level federated edge learning [70], multi-model federated learning [71], and multi-participant multi-class vertical federated learning (MMVFL) [72], all specifically designed for multi-class classification tasks. Despite the breadth of research in federated learning, there remains a notable gap in the literature regarding multi-label-based federated learning approaches, indicating an area ripe for further exploration and development, particularly valuable in animal-related scenarios where data instances may simultaneously belong to multiple classes. 

Multi-label learning challenges the traditional notion of assigning items to a single class and allows items to belong to multiple classes at the same time. This distinction underscores the complexity of classification tasks in modern data analysis. While single-label classification remains fundamental, multi-label classification has emerged as a crucial technique in various domains [73]. However, achieving high accuracy in multi-label classification presents a formidable hurdle, as accurately predicting multiple labels for each item demands sophisticated algorithms. Researchers have offered diverse solutions to handle the intricacies of multi-label classification tasks, including binary relevance (BR) [74], which treats each label as a separate binary classification task, and label powerset (LP) [75], which considers each unique combination of labels as a single class. Classifier chains (CCs) [76] sequentially train multiple binary classifiers, while random k-labelsets (RAkELs) [77] randomly partition the label space into subsets for classification. The ensemble of classifier chains (ECC) [78] combines multiple classifier chains for improved performance.

The multi-label k-nearest neighbors (ML-kNNs) method [79] adapts the k-nearest neighbor algorithm for multi-label classification. Pairwise coupling (PC) [80] trains a binary classifier for each pair of labels, while the majority of label sets [81] predict the most frequent label subset among training instances. Deep learning architectures, such as convolutional neural networks (CNNs) [82], recurrent neural networks (RNNs) [83], and graph neural networks (GNNs) [84], are powerful tools designed specifically for multi-label classification tasks. Additionally, hybrid approaches integrate various techniques to leverage the strengths of different methods, providing robustness in dealing with multi-label classification problems across diverse domains and related datasets, such as transfer learning-based multi-label classification [85], rule-based multi-label classification (MLC) [86], meta-learning based multi-instance multi-label learning (MetaMIML) [87], multi-label long short-term memory (LSTM) [88], the multi-label generative adversarial network (ML-CookGAN) [89], and so on. By reviewing these varied methodologies, valuable insights are gained into the evolving landscape of multi-label learning research in this study.

Recently, research has demonstrated the effectiveness of the REPtree in various machine learning-based tasks, including the rotational forest and reduced-error pruning trees (RTF-REPTree) approach in forest loss analysis [90], the ensemble models of REPTree in geospatial analysis [91], the combination of REPTree, additive regression (AR), regression by discretization (RD), and random committee (RC) models to predict the quality of river waters [92], the utilization of REPTree for air quality monitoring [93], the employment of REPTree in predicting landslide susceptibility (LSM) [94,95], the social engagement analysis of students during the COVID-19 pandemic through REPTree [96], the REPTree-based estimation of evapotranspiration (ETo) from the reference surface in agricultural planning [97], the enhancement of security in industrial internet of things (IIoT) to mitigate cyber-attacks via the REPTree and other ML algorithms [98], and the analysis of fear-inducing factors using the REPTree in reaction to the omicron variant of the coronavirus amidst academic societies [99]. While numerous types of decision trees, including GBDT [100,101,102,103,104,105,106], XGBoost [107,108,109,110,111,112,113,114,115,116,117], RF [118,119,120], and Extra Trees [121], have been utilized within federated learning methods, the literature notably lacks references to the REPtree. Renowned for its proficiency in handling noisy data and its interpretability, the REPtree holds promise for providing distinct advantages in federated learning.

It is noteworthy to consider that the classification of decision tree aggregation encompasses two primary groups, namely, aggregation decision trees and selecting decision trees, each with distinct methodologies. In the aggregation decision tree category, four types are delineated, including structured-based, weight-based, logic-based, and dataset-based approaches. Structured-based aggregation involves organizing decision trees hierarchically and then amalgamating different layers, thereby classifying samples within sub-nodes based on this hierarchical structure. Weight-based aggregation comprises treating divisions within the tree as sets and aggregating the weight values associated with samples in each set. Logic-based aggregation constructs decision trees as sets of logical rules, subsequently aggregating the logical expressions derived from these rules. Dataset-based aggregation entails fitting the outcomes of multiple decision trees onto a comprehensive dataset. In contrast, choosing decision trees involves iteratively selecting a single tree that optimally encapsulates the information across all the datasets, thereby serving as the global model. This systematic approach for decision tree aggregation and selection facilitates robust modeling across diverse datasets and problem domains [61].

While the REPtree has shown remarkable effectiveness across various machine learning tasks, including those mentioned earlier, its potential within the realm of federated learning and multi-label learning, particularly when combined with the binary relevance approach, remains relatively unexplored. Federated learning, which enables distributed model training across multiple components while keeping data decentralized, presents a powerful framework for effectively integrating algorithms like the REPtree. Similarly, multi-label learning is used to predict multiple labels for a single instance and could benefit from the proficiency of the REPtree. However, the intersection of these fields with the REPtree has yet to be deeply investigated, representing an intriguing avenue for further research in the current study.

## 3. Materials and Methods

### 3.1. Proposed Approach

This paper proposes a federated-learning-based approach that trains data distributed on the nodes and learns a global model by aggregating locally trained models. This innovative strategy aimed to revolutionize the traditional model of machine learning by decentralizing the training process. Instead of gathering user data into a centralized repository, it implements a distributed approach where each device independently trains a predictive model using locally stored data. The central server aggregates local models, refining the predictive capabilities of the model. This innovative technique not only enhances the performance of machine learning applications but also sets a new standard for privacy-preserving machine learning practices in diverse applications and industries.

Federated learning encompasses three primary steps: global model and constraints initialization, local training, and model aggregation. Notably, only the second step belongs to the local participants, while the remaining two are handled on the aggregation server side. Consider synchronized algorithms for federated learning, where a standard round entails the following sequence of steps: Firstly, a subset of clients is selected. Subsequently, each client builds or updates its local model based on its local private data. Then, the local models from these clients are transmitted to the server. Finally, the server aggregates these models to construct an enhanced global model. Hereby, a model resembling a traditionally centralized machine learning model is jointly constructed in an efficient way. Moreover, federated learning offers several notable advantages. Firstly, it enhances data privacy by retaining data on the client, thereby safeguarding sensitive information. Disclosure control mechanisms, such as differential privacy and homomorphic encryption, can be employed to further protect data during the exchange of model updates. Additionally, it enhances efficiency by distributing model training across multiple clients, allowing for parallelized and accelerated learning processes [122].

The federated learning architecture encompasses various approaches tailored to different data distribution scenarios: horizontal federated learning (HFL), vertical federated learning (VFL), and federated transfer learning (FTL). In HFL, local datasets may have the same feature space and different sample spaces. Each node trains a local model using its respective data, and the local models or outputs are then transmitted to a central server. The server aggregates these results and gives a response to the user, facilitating collaborative model training. Conversely, VFL utilizes vertical data partitioning, where the datasets of each client may have the same sample space and different feature spaces. This setup allows the ability to build an accurate model as participants retain their data and models locally, exchanging intermediate computation results with the server. FTL introduces a hybrid approach to data partitioning, characterized by a common sample space and different feature spaces. This setup is particularly useful for scenarios where there is minimal overlap in both data features and data samples among participants. FTL enables knowledge transfer across heterogeneous datasets by leveraging pre-trained models or representations from one domain to enhance learning in another domain, thereby maximizing the utility of disparate data sources [123]. Each federated learning approach offers distinct advantages and is tailored to specific data distribution characteristics, ensuring flexibility and scalability in addressing diverse real-world scenarios while maintaining data privacy and efficiency. In this study, we specifically employed VFL due to its ability to leverage the same sample space with differing target label features, which enriches the information about samples and facilitates the construction of multiple binary classifiers for multiple labels. In other words, this approach ensures that the number of instances for each client is equal, and therefore balanced as well.

In the binary relevance approach, the multi-label problem is decomposed into several binary classification tasks. Here, each label is handled as an independent binary classification task. This means that a separate binary classifier is trained on each client node to predict its presence or absence for a given instance. In other words, the number of client nodes is equal to the number of labels in the dataset. Therefore, label size impacts the addition or removal of client nodes in the final model. Consequently, the output of the binary classifiers is a set of binary predictions, one for each label. In addition to its simplicity, the binary relevance approach offers several advantages. It allows for the utilization of standard binary classifiers, shortens the learning process, and provides interpretability as the prediction of each label is independent of others. However, one potential drawback of the binary relevance approach is that it does not consider the correlations between labels, which could be important in certain applications. While our datasets do not require correlated labels, making this limitation less impactful in our context, it is worth noting for other potential applications. As a solution, the classifier chains method can be employed, which passes label information between classifiers and incorporates label correlations. This approach effectively captures label dependencies and addresses the limitations of the binary relevance method, potentially enhancing performance in scenarios where label correlations are significant.

**Figure 1:**
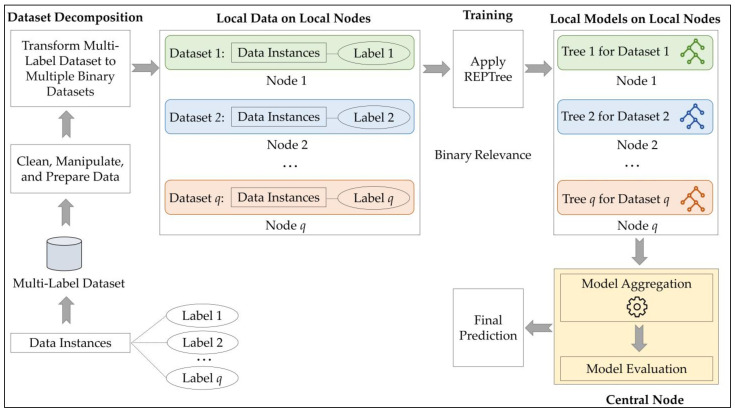
The architecture of the proposed FMLL method.

In the proposed system, as shown in Figure 1, a central node collaborates with several local nodes (or clients) as the standard step of federated learning. In the architecture, the method manages instances with multiple labels, such as label 1 to label *q*, resulting in a multi-label dataset as the input. Initially, preprocessing operations are conducted to clean, manipulate, and prepare the data. Subsequently, dataset decomposition is performed to transform the multi-label dataset into multiple binary datasets, following the binary relevance approach. This decomposition yields datasets 1 to *q*, where instances possess binary labels—for example, dataset 1 indicates whether label 1 exists or not. These transformed datasets serve as local data on local nodes, acting as local clients within the federated learning framework. In the training phase, the REPTree algorithm is applied to each dataset, generating local models on local nodes—tree 1 corresponds to dataset 1, and so forth. Following this, in the central node, local models are aggregated to create a global model. After that, model evaluation takes place, where its performance is assessed using metrics such as accuracy, precision, recall, and F-score. This step ensures that the collective knowledge from the local models is effectively integrated. The final model in the central node facilitates predictions based on the input query data. This integrated approach offers a comprehensive solution for handling multi-label datasets within a federated learning context, providing scalability and efficiency while maintaining model performance.

### 3.2. Formal Description

Traditional supervised learning algorithms operate within the framework of single-label scenarios, where each sample in the training set is related to a sole label defining its characteristics. In contrast, multi-label learning algorithms deal with samples in the training set that are concurrently linked to multiple labels. The objective of multi-label learning is to predict the appropriate label set for unseen samples, which may encompass more than one label per. Here, the definition of multi-label learning is formally established. Given D as the training set comprising N samples $\;S_{i}=(x_{i},Y_{i})$, where  i=1,2,…,N, each sample $S_{i}$ is paired with a feature vector $x_{i}=(x_{i1},x_{i2},\ldots x_{iK})$ having K elements and a subset of labels ${\;Y}_{i}\subseteq L$, where $L=\left\{y_{j}\;\right|\;j=1\;to\;q\}$ represents the set of *q* probable labels. This representation is depicted in Table 2. In this context, the objective of a multi-label learning algorithm is to construct a global model G that, given an unlabeled instance  S=(x,?), precisely predicts its subset of labels  Y, denoted as $\;G\left(S\right)\to Y$, where Y represents the labels associated with the sample  S.

Table 2 illustrates a multi-label dataset where each sample S is associated with a subset of labels denoted by  Y. For instance, $S_{1}$ is associated with the label set $Y_{1}$ containing $y_{2}$ and ${\;y}_{4}$, indicating that this instance possesses both labels $y_{2}$ and ${\;y}_{4}$. It is noteworthy to regard that the outputs from all classifiers are combined with the concatenate operator. Here, the label set$\;Y_{1\;}$includes the concatenation of both labels $y_{2}\;$and ${\;y}_{4}$. Similarly, the sample $S_{2}$ belongs to $\;y_{1}$, $y_{3}$, and $y_{4}$ classes simultaneously, given with a concatenate operator. These representations showcase the multi-label nature of the dataset, where instances may have multiple associated labels simultaneously.

The binary relevance method represents a problem transformation approach that breaks down a multi-label classification task into multiple single-label binary classification problems, each corresponding to one of the q labels in the set $\;L=\{y_{1},y_{2},{\ldots ,y}_{q}\}$. Primarily, this method converts the initial multi-label training dataset into q binary datasets $D_{y_{j}}$, j=1,2,…,q, where $D_{y_{j}}$ encompasses all samples from the initial dataset but with a singular positive or negative label attributed to the label $y_{j}$ based on the true label subset related to each sample. In essence, a label is considered positive if it is included in the label set containing $\;y_{j}$; if not, it is considered negative. Following this transformation of the multi-label data, a collection of q binary classification models ${\;M}_{j}$, where  j=1,2,…,q, is then developed using the respective datasets $D_{y_{j}}$. Finally, the local q models are aggregated to create the global model  G, as indicated by Equation (1):

(1) $$G=\left\{M_{j}\left(\left(x,y_{j}\right)\right)\to {y_{j}}^{\prime }\in \left\{0,1\right\}\;|\;y_{j}\in L\;:j=1...q\right\}$$

To elucidate the fundamental concept of the binary relevance transformation procedure, Table 3 showcases the four binary datasets formed subsequently to transform the multi-label dataset as depicted in the preceding Table 2. In this context, the class attribute can take on two potential values: “present”, denoted as $y_{j}$, or “not present”, represented as $\neg y_{j}$. Each row in Table 3 corresponds to a sample $\left(S_{1},S_{2},\ldots ,S_{N}\right)$ from the original dataset, while each target column represents a distinct label $\left(y_{1},y_{2},y_{3},y_{4}\right)$. Through this transformation, the binary datasets are constructed by discerning the presence or absence of individual labels for each sample. For instance, the positive indicators ($y_{j}$) signify the presence of a label, while negative indicators (${\neg y}_{j}$) indicate its absence. By comparing Table 3 with Table 2, it becomes evident how the labels associated with each example are encoded into binary attributes, simplifying the classification task. For instance, $S_{2}$ in Table 2 is associated with $y_{1}$, $y_{3}$, and $y_{4}$, which is reflected in Table 3 by the presence of $y_{1}$, $y_{3}$, and $y_{4}$, respectively, and the absence of ${\neg y}_{2}$. This transformation facilitates the utilization of conventional binary classification algorithms to handle multi-label classification tasks more effectively. 

The Binary Relevance (BR) method is employed to classify new multi-label samples by aggregating labels positively identified by independent binary classifiers. An inherent advantage of the BR approach lies in its low computational complexity relative to other multi-label methods. Specifically, for a fixed number of samples, the scalability of BR is directly proportional to the size (q) of the label set (L). Given that the complexity of the base classifiers is constrained to  O(C), the overall complexity of BR becomes  q∗O(C). As a result, the BR method proves to be particularly suitable for scenarios where the value of q is not excessively large. However, given the prevalence of numerous labels across various domains, alternative methods, such as divide and conquer approaches, have emerged to establish labels into a tree-shaped hierarchy, allowing for the management of a substantially smaller set of labels in comparison with  q.

Algorithm 1 is devised to address the Federated Multi-Label Learning (FMLL) method through a structured approach divided into two main phases. The client learning process begins with data preparation, given the dataset  D  comprising  N  instances represented as $\;(x_{i},Y_{i})$, where $\;x_{i\;}$ denotes the feature vector and $\;Y_{i\;}$ represents the associated labels, along with  q  as the number of nodes (or the number of class labels) and ${\;M}_{j}$ as the local models for each label. The dataset *D* is partitioned into q binary datasets based on the presence of each class label ${\;y}_{j}$. Each node generates local datasets $\;D_{y_{j}}$, marking instances as 1 if ${\;y}_{j}\;$ is present in ${\;Y}_{i}\;$ and 0 otherwise, and stores them locally. Subsequently, in local model training, each node independently trains local models ${\;M}_{j}\;$using the REPTree algorithm on their respective binary datasets ${\;D}_{y_{j}}$. These trained models ${\;M}_{j}\;$ are then transmitted to the central server for further processing. The server aggregation process integrates the received local models ${\;M}_{j}\;$ to construct a unified global model  G  through the model aggregation approach. The central server combines these models to form  G, representing a comprehensive synthesis of knowledge from all nodes. Using this global model, the algorithm performs classification tasks on the test set  T. For each instance  x  in  T, predictions are made by aggregating outputs from all local models, resulting in the final predicted label set $\hat{\;Y}$. Thus, the algorithm provides a systematic approach to federated multi-label learning by incorporating distinct client learning and server aggregation processes. This structured methodology ensures robustness and reproducibility in handling distributed datasets and synthesizing global models, essential for effective multi-label prediction across decentralized environments.
**Algorithm 1:** Federated Multi-Label Learning (FMLL)1. Client Learning Process Inputs:   D: dataset D
$={\left\{\left(x_{i},Y_{i}\right)\right\}}_{i=1}^{N}$
  q
*=* number of nodes (number of class labels at the same time) Outputs:  $M_{j}$: local models for each label1.1. Data Preparation Begin   for j
*=* 1 to q     foreach $\left(x_{i},Y_{i}\right)$ in D     // Generate binary datasets       if ($y_{j}\in Y_{i}$)         $D_{y_{j}}.Add(x_{i},1)$       else         $D_{y_{j}}.Add(x_{i},0)$       endif     end foreach      Store ($D_{y_{j}})$           // Store local data at the node *j*  end for 1.2. Local Model Training  for j
*=* 1 to q
    $M_{j}$ = $REPTree(D_{y_{j}})$     // Train local models at each node in parallel     Send ($M_{j}$)            // Send $M_{j}\;$ to the central server  end for End2. Server Aggregation Process Inputs:  $M_{j}:\;$local models from each client for each class label  q
*=* number of nodes (number of class labels at the same time)  T: test set that will be predicted Outputs:  G: global model   $\hat{Y}:\;$predicted labels for test set2.1. Model Aggregation Begin   G= Ø   for j
*=* 1 to q     Receive ($M_{j}$)   // Receive local models $M_{j}\;$ from each client     G = $G\;\cup M_{j}$   // Aggregate the models to form the global model  G  end for 2.2. Classification   foreach x in T
     for j
*=* 1 to q      y = G(x)
      $\hat{Y}$ = $\hat{Y}\cup y$  //Predict y using the global model G     end for  end foreach End

## 4. Experimental Studies

### 4.1. Dataset Description

The study of animals in their natural habitats is fundamental to our understanding of ecological dynamics, biodiversity conservation, and species management. Animal behavior, physiology, and interactions with their environment provide invaluable insights into the functioning of ecosystems and the intricate balance of life on our planet. In this paper, we harness the richness of animal-related datasets to evaluate the efficacy of our proposed Federated Multi-Label Learning (FMLL) method within the vibrant field of animal research. Table 4 provides a summarized overview of these datasets utilized in the current study. In this table, the respective number of classes is represented for each label in the datasets.

#### 4.1.1. Amphibians

The Amphibians Habitat Classification dataset, briefly presented in Table 5, is collected from a combination of geographic information systems (GIS), satellite imagery, and field inventories conducted as part of environmental impact assessments (EIAs) for two planned road projects, including Road A and Road B in Poland [124]. Amphibians, as crucial animal indicators of environmental health and ecosystem integrity due to their sensitivity to environmental changes, play a vital role in assessing the impact of infrastructure projects on biodiversity, particularly within their habitat. Integrating GIS and satellite information with data collected from natural inventories, field research was directed within a 500-m-wide strip on both sides of the proposed project area for Road A, identifying 80 amphibian breeding sites, while Road B’s inventory focused on the vicinity of two variants of the planned Beskidy Integration Way, covering approximately 60 km and resulting in the identification of 109 amphibian occurrence sites through map analysis, field observations, a literature review, and archive data analysis. The dataset comprises multiple variables, contributing to a comprehensive understanding of amphibian habitats within the realm of biology. It was primarily generated for classification tasks, capturing diverse environmental characteristics relevant to amphibian habitat suitability.

This multivariate dataset with 189 samples and 23 features provides valuable insights into the ecological implications of road infrastructure development on amphibian populations, facilitating biodiversity conservation and informed decision-making in environmental management with the aim of predicting the existence of seven different animals, namely green frogs, brown frogs, common toads, fire-bellied toads, tree frogs, common newts, and great crested newts with labels one to seven, respectively. The dataset encompasses three distinct numerical features, as detailed in Table 6, showcasing their statistical attributes such as minimum, mean, maximum, mode, and standard deviation. Additionally, Table 7 comprehensively explains all features, providing deeper insight into the instances collected.

#### 4.1.2. Anuran-Calls-(MFCCs)

The Anuran-Calls-(MFCCs) dataset [125] comprises acoustic features extracted from syllables of anuran (frogs) calls, accompanied by multi-label annotations indicating their family, genus, and species, as represented in Table 8. With a total of 7195 instances, this multivariate dataset has been extensively utilized in various classification and clustering tasks, particularly within the realm of biology. Furthermore, the dataset incorporates 22 separate numerical features, elaborated in Table 9, and highlights their statistical characteristics, including maximum, minimum, mean, mode, and standard deviation. Its completeness and reliability are attributed to the absence of missing values, markedly enhancing its suitability for such analytical endeavors.

The Anuran-Calls-(MFCCs) dataset originates from the segmentation of 60 audio recordings spanning four distinct families, eight genera, and ten species of anuran frogs. Each audio recording corresponds to a single specimen, with an additional record ID column included for reference. The distribution of instances for each family, genus, and species class is given in Table 10. The recordings were conducted in situ under real noise conditions, capturing the natural background sounds, thereby offering a diverse representation of anuran habitats, including locations such as the campus of the Federal University of Amazonas in Manaus, the Mata Atlantic region in Brazil, and even one location in Córdoba, Argentina. Recorded in WAV format at a sampling frequency of 44.1 kHz and a 32-bit resolution, the dataset enables signal analysis up to 22 kHz. The feature extraction process involved calculating 22 Mel-Frequency Cepstral Coefficients (MFCCs) for each syllable, employing 44 triangular filters. These coefficients are subsequently normalized within the range of −1 to 1 and are statistically discussed in Table 9.

The Anuran-Calls-(MFCCs) dataset, with its rich acoustic features and multi-label annotations, is a valuable asset for advancing research in anuran species recognition and related fields. Anurans play crucial roles in ecosystems worldwide, serving as indicators of ecosystem health and biodiversity. They regulate populations of insects and other invertebrates, maintaining ecological balance within animal food webs. Additionally, their skin contains bioactive compounds with potential pharmaceutical applications, contributing to medical research. However, anuran species are threatened by habitat destruction, pollution, and climate change, requiring robust analysis and conservation efforts. Furthermore, they are important for education and outreach initiatives, promoting public awareness of ecology, biodiversity, and conservation.

#### 4.1.3. HackerEarth-Adopt-A-Buddy 

The HackerEarth-Adopt-A-Buddy dataset [126] served a noble purpose in facilitating the creation of a virtual tour experience for an esteemed pet adoption agency amidst the pandemic, introduced in Table 11. As the pandemic saw a surge in animal adoption and fostering, this initiative aimed to keep potential pet owners engaged indoors by virtually presenting animals accessible for adoption. To support this endeavor, machine learning methods can be developed to determine the type and breed of animals based on their physical attributes and other pertinent factors. The description of all features in the HackerEarth-Adopt-A-Buddy dataset is summarized in Table 12. The dataset provides a comprehensive foundation for predictive model development and evaluation with 18,834 entries in the training dataset. Moreover, within the dataset, there are four distinct numerical features outlined in Table 13, presenting their statistical attributes such as minimum, maximum, mean, mode, and standard deviation.

This dataset presents an opportunity for multi-label classification as a fundamental aspect of machine learning. By utilizing the provided data and employing machine learning techniques, researchers are tasked with constructing a predictive model capable of accurately discerning both the breed category and pet category based on factors such as animal condition, appearance, and other relevant attributes. This dataset contributes to the important cause of promoting pet adoption and fostering. 

Pets serve a crucial role in animal science, offering researchers invaluable insights into various aspects of behavior, physiology, and health. Beyond companionship, they provide real-life settings for studying topics such as animal nutrition, genetics, psychology, and disease management. Moreover, pets serve as models for understanding human–animal interactions, leading to advancements in veterinary medicine and animal welfare. Studying pets yields insights that benefit both human and animal well-being, making them indispensable in the field. Additionally, pet adoption holds significant importance in animal science, extending beyond providing loving homes for animals in need. It serves as a vital avenue for research and education within the discipline. Researchers gain valuable insights into behavior, health, and welfare by studying adopted animals in diverse environments. The diversity among adopted animals allows for the exploration of genetic variations and their impacts on traits and diseases, contributing to veterinary medicine and animal breeding practices. Furthermore, the adoption process fosters public awareness and appreciation for animal welfare issues, promoting responsible pet ownership and ethical treatment. Embracing pet adoption not only enriches individual lives but also advances our understanding and care of the animal kingdom through the analysis of related datasets.

### 4.2. Results 

The primary objective of this study is to introduce an innovative method termed Federated Multi-Label Learning (FMLL) designed specifically for classification tasks. By integrating insights from well-established methodologies such as Binary Relevance and the Reduced-Error Pruning Tree (REPTree) approaches, our framework seeks to synergize the advantages of federated learning and multi-label concepts. This integration is aimed at tackling the complexities associated with multi-label classification issues. The efficacy of the FMLL method was validated using dedicated multi-label datasets, including Amphibians, Anuran-Calls-(MFCCs), and HackerEarth-Adopt-A-Buddy. Our approach was implemented in the C# programming language utilizing the Weka library [127]. The source codes of both FMLL and REPTree methods are publicly available in the GitHub archive (https://github.com/BitaGhasemkhani/Federated-Multi-Label-Learning-FMLL, accessed on 28 June 2024), ensuring reproducibility. 

The REPTree classifier was used in our experiments, with hyperparameters set to their default values, e.g., batchSize (100), debug (False), doNotCheckCapabilities (False), initialCount (0.0), maxDepth (−1), minNum (2.0), minVarianceProp (0.001), noPruning (False), numDecimalPlaces (2), numFolds (3), seed (1), and spreadInitialCount (False). The experiments were conducted on standard machines (i.e., Intel(R) Core(TM) i5, 1.80 GHz, 4.00 GB RAM). Also, we employed the 10-fold cross-validation method during experimentation to train and assess the classification models. This method involves randomly dividing the dataset into ten sets, reserving one set for testing while the remaining nine sets serve as the training set. The evaluation process was iterated ten times, and the average classification accuracy was computed.

Furthermore, we employed a range of metrics to evaluate the performance of the proposed FMLL method, including accuracy (ACC), precision (PR), recall (TPR), F-score (FS), and true negative rate (TNR) as delineated in Equations (2) to (6). Moreover, we used the receiver operating characteristic (ROC) curve to assess the trade-off between the true positive rate (TPR) from Equation (4) and the false positive rate (FPR) from Equation (7). Additionally, the precision–recall curve (PRC) was utilized to evaluate the balance between precision and recall.

(2) $$ACC=\frac{TP+TN}{TP+TN+FP+FN}$$

(3) $$PR=\frac{TP}{TP+FP}$$

(4) $$TPR=\frac{TP}{TP+FN}$$

(5) $$FS=\frac{2TP}{2TP+FP+FN}$$

(6) $$TNR=\frac{TN}{TN+FP}$$

(7) $$FPR=\frac{FP}{FP+TN}$$

In this context:

- True Positive (TP) signifies the count of correctly predicted positive classes by the classifier.
- True Negative (TN) represents the count of accurately predicted negative classes by the classifier.
- False Positive (FP) denotes the count of erroneously predicted positive classes by the classifier.
- False Negative (FN) indicates the count of erroneously predicted negative classes by the classifier.

The application of Federated Multi-Label Learning (FMLL) to the Amphibians dataset yielded compelling results, as shown in Table 14, achieving an average accuracy of 73.24%. Precision scores ranged from 0.613 to 0.790, while recall scores varied from 0.656 to 0.884, demonstrating FMLL’s effectiveness in accurately classifying various amphibian species. Moreover, the F-score, ranging from 0.619 to 0.834, underscored the method’s capability to manage dataset complexities while maintaining a balanced performance between precision and recall. The ROC curve results, spanning from 0.503 to 0.715, highlighted variable performance in class differentiation, whereas the PRC values, ranging from 0.603 to 0.818, provided valuable insights into precision–recall trade-offs across different thresholds. Additionally, the TNR scores between 0.655 and 0.884 indicated the method’s reliability in correctly identifying negative instances. Remarkably, the “great crested newt” amphibian emerged as the top performer across all the metrics, except ROC. 

Regarding the Anuran-Calls-(MFCCs) dataset, FMLL showcased exceptional performance, as represented in Table 15, boasting an average accuracy of 94.50%. Precision scores consistently surpassed 0.935 for family, genus, and species categories, demonstrating FMLL’s precision in classifying different levels of anuran calls. Additionally, recall scores ranged from 0.936 to 0.957, underscoring the method’s success in retrieving relevant instances for each category. The F-score, averaging 0.944, further validated FMLL’s effectiveness in handling multi-label classification tasks with high accuracy and reliability. Outstandingly, the “family” syllabus of Anurans excelled in all metrics, achieving an accuracy of 95.75%, with precision, TNR, ROC, PRC, recall, and F-score all reaching above 0.957. Moreover, TNR scores across all categories were considerably high, ranging from 0.980 to 0.992, indicating FMLL‘s ability to accurately identify negative instances. The ROC curve values, ranging from 0.978 to 0.983, illustrated strong performance in distinguishing between classes, while PRC values, ranging from 0.935 to 0.964, offered a detailed analysis of precision–recall dynamics across varying thresholds.

FMLL demonstrated remarkable performance on the HackerEarth-Adopt-A-Buddy dataset, as shown in Table 16, accurately predicting breed and pet categories with an average accuracy of 86.12%. According to the results, the “pet_category” exhibited slightly superior performance compared to the “breed_category” across all the metrics, except ROC and PRC. Also, precision, TNR, ROC, PRC, recall, and F-score metrics presented high average values of 0.863, 0.928, 0.956, 0.933, 0.861, and 0.858, respectively. Furthermore, the ROC values for both categories demonstrated strong discrimination between classes, with values of 0.965 for breed and 0.946 for pet categories. Furthermore, the PRC values, at 0.938 for the breed and 0.928 for pet categories, provided detailed visions into the model‘s precision–recall dynamics. FMLL reaffirmed its robustness in handling complex multi-label classification tasks across different datasets.

**Figure 2:**
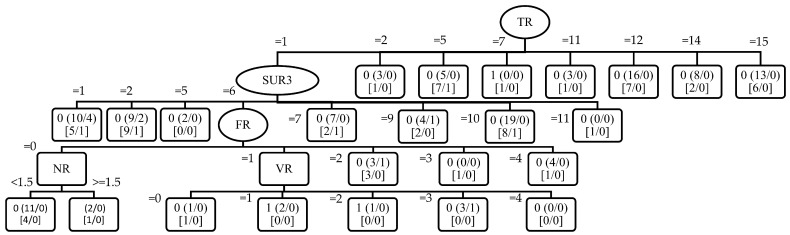
REPTree structure for “great crested newt” classification in FMLL.

As evidenced by Table 14, the FMLL method achieved the highest accuracy (88.36%) on the “great crested newt” species among all the considered metrics. To elucidate the decision-making process underlying this performance, the FMLL method employed a REPTree classifier, generating a structured tree representation as shown in Figure 2. This REPTree structure prominently featured attributes such as type of water reservoirs (TR), surroundings 3 (SUR3), presence of fishing (FR), number of water reservoirs (NR), and vegetation presence (VR) as pivotal nodes. The hierarchical arrangement facilitated a detailed comprehension of feature interactions and their impact on species classification. This illustrative tree not only aids in interpreting model decisions but also underscores the importance of feature selection and attribute significance in FMLL-based classification tasks.

To elaborate further on Figure 2, the root node, labeled TR, represents the most significant attribute for splitting the data, with branches indicating different values of TR. Internal nodes such as SUR3, FR, NR, and VR are actually decision points where data are further split based on specific attribute values. Each leaf node provides the final classification outcome and contains two sets of numbers: (*a*/*b*) and [*c*/*d*]. Here, *a* represents the total number of instances reaching the leaf, *b* indicates the number of misclassified instances, *c* denotes the number of instances of the majority class, and *d* shows the number of instances of the minority class. For example, the leaf node 0 (10/4) [5/1] under SUR3 = 1 and TR = 1 indicates that out of 10 instances, 4 were misclassified, with 5 instances in the majority class and 1 in the minority class. Misclassified instances highlight areas where the model‘s predictions do not align with the actual data, aiding in assessing model accuracy. Subtree analysis under nodes like FR = 6 shows further splits based on values of NR, leading to various leaves with their respective instance distributions. To achieve optimal accuracy, parameters such as the minimum number of instances per leaf were fine-tuned in Weka, ensuring the model balances complexity and generalization. This inclusive interpretation of the REPTree figure enhances our understanding of the model‘s performance and data patterns.

## 5. Discussion

In this section, we compare our proposed method with the current state-of-the-art techniques [124,125,128] in the field. Our analysis covers different dimensions, including accuracy metric on the Amphibians dataset and precision, recall, and F-score evaluation metrics on the Anuran-Calls-(MFCCs) dataset, juxtaposed with state-of-the-art methods, represented in Table 17 and Table 18, respectively.

As shown in Table 17, our approach achieved a remarkable 10.92% improvement on average regarding the Amphibians dataset, outperforming the state-of-the-art methods [124,128]. This improvement can be attributed to the combination of FMLL with BR and the REPTree. While the gradient-boosted tree (GBT), random forest (RF), AdaBoost (ADA), decision tree (DT), and partially monotonic decision tree (PMDT) approaches attained moderate accuracy rates ranging from 57.54% to 71.50%, the proposed method surpassed all these state-of-the-art techniques with the highest accuracy rate of 73.24%. These outcomes highlight the superior performance of FMLL in accurately classifying instances within the multi-label Amphibians dataset.

Table 18 presents a comprehensive comparison of precision, recall, and F-score metrics for various methods using the Anuran-Calls-(MFCCs) dataset, categorized into different taxonomic levels, including species, family, genus, and their combination. At the species level, the FMLL method outperformed all others [125] with precision, recall, and F-score scores of 0.935, 0.936, and 0.935, respectively. The previous methods, e.g., KNN-Flat, RBF-SVM-Flat, Polynomial-SVM-Flat, and Tree-Flat [125], displayed precision scores ranging from 0.470 to 0.850, recall scores ranging from 0.500 to 0.760, and F-scores ranging from 0.490 to 0.740. At the family level, FMLL again revealed superior performance, boasting precision, recall, and F-scores of 0.957 each, outperforming the baseline method, KNN-LCPL. Similarly, at the genus level, FMLL exhibited substantial enhancements over its counterpart, achieving precision, recall, and F-scores of 0.941, 0.942, and 0.941, respectively. Across all taxonomic levels, our method consistently outperformed KNN-LCPL, showcasing precision, recall, and F-scores of 0.944, 0.945, and 0.944, respectively. It is notable that the FMLL method attained substantial improvements across various taxonomic levels when compared to state-of-the-art peers. Specially, at the species taxonomic level, FMLL demonstrated improvements of 25.1% in precision, 30.1% in recall, and 28.4% in F-score metrics. Moving to the family taxonomic level, the method presented improvements of 24.4%, 13.7%, and 19.4% in precision, recall, and F-score metrics, respectively. Similarly, at the genus taxonomic level, FMLL achieved improvements of 27.8%, 21.1%, and 24.6% in precision, recall, and F-score metrics. Finally, when considering the combination of species, family, and genus taxonomic levels, FMLL exhibited improvements of 25.5%, 18.8%, and 22.3% in precision, recall, and F-score metrics. These results underscore the effectiveness of the FMLL method across multiple taxonomic levels, demonstrating substantial improvements over baseline methods in terms of precision, recall, and F-score metrics.

The accuracy results of the existing KNN-LCPL method and the proposed FMLL method given in Table 18 were evaluated using the Mann–Whitney-U and the Quade tests in detail. The Mann–Whitney-U and Quade as non-parametric statistical tests are ideal for comparing the performances of algorithms, making them appropriate for our analysis. The obtained *p*-values from the Mann–Whitney-U and Quade tests are 0.02107 and 0.03047, respectively. These results show that *p*-values are considerably below the significance level of 0.05 (α = 0.05). These results indicate that the likelihood of the results occurring by random chance is minimal, allowing us to reject the null hypothesis, which suggests no difference in performance between the methods. Therefore, these statistical tests provide strong evidence that the proposed FMLL method significantly outperformed the KNN-LCPL method. The very small *p*-values obtained underscore the substantial and reliable differences in accuracy between the two methods.

## 6. Conclusions and Future Work

In summary, this study introduces Federated Multi-Label Learning (FMLL) as a groundbreaking approach in animal science classification to address the challenges posed by distributed data. By blending federated learning principles with multi-label learning techniques, FMLL offers a method for handling classification tasks where instances may belong to multiple classes simultaneously. Utilizing the Binary Relevance (BR) strategy and adopting the Reduced-Error Pruning Tree (REPTree) classifier within the federated learning framework, FMLL demonstrated robust performance and showcased significant improvements (above 10%) in classification accuracy across diverse animal species datasets. Empirical validation on three distinct datasets—Amphibians, Anuran-Calls-(MFCCs), and HackerEarth-Adopt-A-Buddy—underscored the effectiveness of FMLL in real-world scenarios. Notably, the classification accuracy reached 94.50% for the Anuran-Calls-(MFCCs) dataset and 86.12% for the HackerEarth-Adopt-A-Buddy dataset, highlighting the robustness and practical relevance of FMLL across various taxonomic levels and its potential for applications in diverse domains. Having explored the advancements and contributions of the current research, the following conclusions highlight the significant impacts of the proposed method on the field of animal studies:

(i) Introduction of FMLL (with BR and REPTree) in animal science classification as a novel approach, applicable to diverse real-world scenarios.
(ii) Providing the distribution of computational cost over several clients and ensuring data security with FMLL to preserve privacy in collaborative learning environments.
(iii) Effective handling of multi-label data within the FMLL framework using the BR strategy.
(iv) Pioneering use of the REPTree classifier in federated learning, enhancing accuracy and interpretability.
(v) Empirical validation of FMLL on various animal-based datasets, demonstrating its reliable applicability and efficacy in the field.
(vi) The superiority of FMLL in multi-label classification tasks, evidenced by higher accuracy, precision, recall, and F-score metrics compared to state-of-the-art methods.
(vii) The practical relevance of FMLL across taxonomic levels, showcasing its reliability in addressing multi-label classification problems within the context of animal research.

Looking ahead, several avenues emerge for further exploration of FMLL. Firstly, developing a web application that provides an interface to access the FMLL-based machine-learning model could be useful for animal scientists in decision-making. Additionally, extending FMLL to accommodate dynamic datasets collected by IoT devices, along with integrating mechanisms for model updating, could bolster its adaptability and long-term performance. Exploring alternative multi-label learning methodologies, such as classifier chains, would address the current limitation of binary relevance by incorporating label correlations. Moreover, ensemble learning techniques could be further integrated with FMLL by combining predictions from multiple models. Further exploration of deep learning architectures within the FMLL framework presents an opportunity to uncover profound insights into complex patterns inherent in animal science data. By focusing on these research directions, we aspire to propel the field of federated multi-label learning forward and advance its applications in animal science classification tasks.

## Figures and Tables

**Table 1:** Overview of federated learning frameworks.

Year	Ref.	FL Type	Dataset	Aggregation Algorithm	ML Algorithm	Evaluation Metric	Contribution
2023	[52]	Centralized	Air pollutants and meteorolgical data	FedAvg	LSTM, SSA, and DPLA	MAE, RMSE, R-squared	Cross-domain prediction of air pollutant concentration
2023	[53]	Decentralized	Air dataset	FedAvg	CNN	Accuracy, precision, recall, F-score, and confusion matrix	Predicting chickpea crops for smart farming
2023	[54]	Centralized	Crop and soil dataset	Federated learning	AMFSC	Analysis rate, control rate	Agricultural production improvement
2022	[30]	Centralized	CSE-CICIDS2018, MQTTset, and InSDN	Cyber-physical production system (CPPS)-based aggregation	CNN, recurrent neural networks, and deep neural networks	Accuracy, precision, recall, F-score	Intrusion detection to enhance the security of agricultural IoT infrastructures
2022	[55]	Centralized	Wind turbines data	FedAvg	MSRAN and deep network	Precision, recall, and F-score	Fault detection in wind turbines
2022	[56]	Centralized	ToN_IoT	FedAvg and Fed+	Multinomial logistic regression	Accuracy, precision, recall, F-score, FPR	Intrusion detection for IoT
2022	[57]	Centralized	Spinesagt2- wdataset3	Federated contrastive learning optimization (FCLOpt)	Dual attention gates (DAGs) and U-Net	Accuracy	Federated learning-based vertebral body segment framework (FLVBSF)
2022	[58]	Centralized	N-BaIoT	Mini-batch and multi-epoch aggregation, derived from FedAVG	Multilayer perceptron and autoencoder	Accuracy, F-score	Federated learning for IoT malware detection
2022	[59]	Centralized	SWAT 2015	SCADA server-based aggregation	GBDT with Paillier HE	Accuracy	Intrusion detection for IoT prioritizing data confidentiality
2021	[60]	Decentralized	CWRU	FA-FedAvg	CNN	Accuracy	Bearing fault diagnosis

**Table 2:** Example representation of instances in multi-label learning.

Sample	X				Y
$S_{1}$	$x_{11}$	$x_{12}$	…	$x_{1K}$	$Y_{1}=\{y_{2},y_{4}\}$
$S_{2}$	$x_{21}$	$x_{22}$	…	$x_{2K}$	$Y_{2}=\{y_{1},y_{3},y_{4}\}$
…	…	…	…	…	…
$S_{N}$	$x_{N1}$	$x_{N2}$	…	$x_{NK}$	$Y_{N}=\{y_{3}\}$

**Table 3:** Binary Relevance transformation of the multi-label dataset displayed in Table 2.

$D_{y_{1}}$	X	Y	$D_{y_{2}}$	X	Y	$D_{y_{3}}$	X	Y	$D_{y_{4}}$	X	Y
$S_{1}$	$\left[x_{11}\ldots x_{1K}\right]$	${\neg y}_{1}$	$S_{1}$	$\left[x_{11}\ldots x_{1K}\right]$	$y_{2}$	$S_{1}$	$\left[x_{11}\ldots x_{1K}\right]$	${\neg y}_{3}$	$S_{1}$	$\left[x_{11}\ldots x_{1K}\right]$	$y_{4}$
$S_{2}$	$\left[x_{21}\ldots x_{2K}\right]$	$y_{1}$	$S_{2}$	$\left[x_{21}\ldots x_{2K}\right]$	$\neg y_{2}$	$S_{2}$	$\left[x_{21}\ldots x_{2K}\right]$	$y_{3}$	$S_{2}$	$\left[x_{21}\ldots x_{2K}\right]$	$y_{4}$
…	…	…	…	…	…	…	…	…	…	…	…
$S_{N}$	$\left[x_{N1}\ldots x_{NK}\right]$	${\neg y}_{1}$	$S_{N}$	$\left[x_{N1}\ldots x_{NK}\right]$	${\neg y}_{2}$	$S_{N}$	$\left[x_{N1}\ldots x_{NK}\right]$	$y_{3}$	$S_{N}$	$\left[x_{N1}\ldots x_{NK}\right]$	${\neg y}_{4}$

**Table 4:** A brief overview of utilized datasets.

ID	Ref.	Dataset Name	#Features	#Instances	#Labels	#Classes	Source	Link (accessed on 16 March 2024)
1	[124]	Amphibians	23	189	7	2,2,2,2,2,2,2	UCI	https://archive.ics.uci.edu/dataset/528/amphibians
2	[125]	Anuran-Calls-(MFCCs)	22	7195	3	4,8,10	UCI	https://archive.ics.uci.edu/dataset/406/anuran+calls+mfccs
3	[126]	HackerEarth-Adopt-A-Buddy	11	18,834	2	3,4	Kaggle	https://www.kaggle.com/datasets/mannsingh/hackerearth-ml-challenge-pet-adoption

**Table 5:** The information of Amphibians dataset.

Dataset Attributes	Task	Study Domain	Feature Types	#Instances	#Features	#Views
Multivariate	Classification	Biology	Integer, real, nominal	189	23	6457

**Table 6:** The statistics of numerical features in Amphibians dataset.

Feature Name	Min	Max	Mean	Mode	Standard Deviation
SR	30	500,000	9633.2275	300	46,256.0783
NR	1	12	1.5661	1	1.5444
OR	25	100	90.8689	100	19.0996

**Table 7:** The description of all features in Amphibians dataset.

No	Attribute	Type	Description
1	ID	Integer	Identification number (unused in classification)
2	MV	Categorical	Motorway (unused in classification)
3	SR	Numerical	Surface of water reservoir (m^2^)
4	NR	Numerical	Number of water reservoirs in habitat (The greater the number of reservoirs, the higher the probability that some of them will be proper for amphibian breeding).
5	TR	Categorical	Type of water reservoirs (including reservoirs with natural features, lately formed reservoirs, settling ponds, reservoirs situated near residential areas, technological water reservoirs, etc.)
6	VR	Categorical	Vegetation presence within the reservoirs (including absence of vegetation, sparse patches at the edges, densely overgrown areas, abundant vegetation within the reservoir, reservoirs entirely overgrown, etc.)
7	SUR1	Categorical	Surroundings 1 (the predominant land cover types surrounding the water reservoir)
8	SUR2	Categorical	Surroundings 2 (the second most prevalent types of land cover surrounding the water reservoir)
9	SUR3	Categorical	Surroundings 3 (the third most predominant types of land cover surrounding the water reservoir)
10	UR	Categorical	Use of water reservoirs (unused by humans, recreational and scenic use, economic utilization, technological purposes)
11	FR	Categorical	The presence of fishing (limited or occasional fishing, intensive fishing, breeding reservoirs)
12	OR	Numerical	Degree of access from reservoir edges to undeveloped areas: no access, limited access, moderate access, extensive access to open space
13	RR	Ordinal	Minimum distance from the water reservoir to roads categorized as: <50 m, 50–100 m, 100–200 m, 200–500 m, 500–1000 m, >1000 m
14	BR	Ordinal	Building development as minimum distance to buildings <50 m, 50–100 m, 100–200 m, 200–500 m, 500–1000 m, >1000 m
15	MR	Categorical	Maintenance status of the reservoir (including clean, slightly littered, reservoirs heavily or very heavily littered)
16	CR	Categorical	Type of shore (natural or concrete)
17	Green frogs	Categorical	Presence of green frogs (label 1)
18	Brown frogs	Categorical	Presence of brown frogs (label 2)
19	Common toad	Categorical	Presence of common toad (label 3)
20	Fire-bellied toad	Categorical	Presence of fire-bellied toad (label 4)
21	Tree frog	Categorical	Presence of tree frog (label 5)
22	Common newt	Categorical	Presence of common newt (label 6)
23	Great crested newt	Categorical	Presence of great crested newt (label 7)

**Table 8:** The information of Anuran-Calls-(MFCCs) dataset.

Dataset Attributes	Task	Study Domain	Feature Type	#Instances	#Features	#Views
Multivariate	Classification, clustering	Biology	Real	7195	22	5692

**Table 9:** The statistics of MFCC syllables in Anuran-Calls-(MFCCs) dataset.

Feature Name	Min	Max	Mean	Mode	Standard Deviation
MFCCs_1	−0.2512	1.0000	0.9899	1.0000	0.0690
MFCCs_2	−0.6730	1.0000	0.3236	1.0000	0.2187
MFCCs_3	−0.4360	1.0000	0.3112	1.0000	0.2635
MFCCs_4	−0.4727	1.0000	0.4460	1.0000	0.1603
MFCCs_5	−0.6360	0.7522	0.1270	No	0.1627
MFCCs_6	−0.4104	0.9642	0.0979	No	0.1204
MFCCs_7	−0.5390	1.0000	−0.0014	No	0.1714
MFCCs_8	−0.5765	0.5518	−0.0004	No	0.1163
MFCCs_9	−0.5873	0.7380	0.1282	No	0.1790
MFCCs_10	−0.9523	0.5228	0.0560	No	0.1271
MFCCs_11	−0.9020	0.5230	−0.1157	No	0.1868
MFCCs_12	−0.7994	0.6909	0.0434	No	0.1560
MFCCs_13	−0.6441	0.9457	0.1509	No	0.2069
MFCCs_14	−0.5904	0.5757	−0.0392	No	0.1525
MFCCs_15	−0.7172	0.6689	−0.1017	No	0.1876
MFCCs_16	−0.4987	0.6707	0.0421	No	0.1199
MFCCs_17	−0.4215	0.6812	0.0887	No	0.1381
MFCCs_18	−0.7593	0.6141	0.0078	No	0.0847
MFCCs_19	−0.6807	0.5742	−0.0495	No	0.0825
MFCCs_20	−0.3616	0.4678	−0.0532	No	0.0942
MFCCs_21	−0.4308	0.3898	0.0373	No	0.0795
MFCCs_22	−0.3793	0.4322	0.0876	No	0.1234

**Table 10:** The distribution of instances per class in Anuran-Calls-(MFCCs) dataset.

Label	Class	#Instances
Family	Bufonidae	68
	Dendrobatidae	542
	Hylidae	2165
	Leptodactylidae	4420
Genus	Adenomera	4150
	Ameerega	542
	Dendropsophus	310
	Hypsiboas	1593
	Leptodactylus	270
	Osteocephalus	114
	Rhinella	68
	Scinax	148
Species	AdenomeraAndre	672
	AdenomeraHylaedactylus	3478
	Ameeregatrivittata	542
	HylaMinuta	310
	HypsiboasCordobae	1121
	HypsiboasCinerascens	472
	LeptodactylusFuscus	270
	OsteocephalusOophagus	114
	Rhinellagranulosa	68
	ScinaxRuber	148

**Table 11:** The information of the HackerEarth-Adopt-A-Buddy dataset.

Dataset Attributes	Task	Study Domain	Feature Type	#Instances	#Features	#Views
Multivariate	Classification	Biology	Integer, real, nominal, temporal	18,834	11	5605

**Table 12:** The description of all features in the HackerEarth-Adopt-A-Buddy dataset.

No.	Attribute	Type	Description
1	pet_id	Integer	A unique identifier is assigned to each animal up for adoption.
2	issue_date	Temporal	The date when the pet was officially taken in by the shelter.
3	listing_date	Temporal	The date and time when the pet became available for adoption at the shelter.
4	condition	Categorical	The health or physical state of the pet upon arrival at the shelter.
5	color_type	Categorical	The color pattern or combination exhibited by the pet.
6	length	Real	The measured length of the pet is typically in meters.
7	height	Real	The measured height of the pet is typically in centimeters.
8	X1	Integer	The value related with the pet.
9	X2	Integer	The other value related with the pet.
10	breed_category	Categorical	The category or classification of the pet’s breed.
11	pet_category	Categorical	The category or species classification of the pet.

**Table 13:** The statistics of numerical features in the HackerEarth-Adopt-A-Buddy dataset.

Feature Name	Min	Max	Mean	Mode	Standard Deviation
length	0.0000	1.0000	0.5026	0.0800	0.2887
height	5.0000	50.0000	27.4488	21.4000	13.0198
X1	0.0000	19.0000	5.3696	0.0000	6.5724
X2	0.0000	9.0000	4.5773	1.0000	3.5178

**Table 14:** Performance metrics for various amphibian species in FMLL.

Amphibians	Accuracy	Precision	TNR	ROC	PRC	Recall	F-Score
Green frogs	68.78	0.694	0.688	0.715	0.682	0.688	0.689
Brown frogs	78.31	0.613	0.783	0.503	0.665	0.783	0.688
Common toad	71.43	0.712	0.714	0.621	0.653	0.714	0.674
Fire-bellied toad	70.37	0.669	0.704	0.576	0.612	0.704	0.650
Tree frog	65.61	0.639	0.655	0.638	0.627	0.656	0.631
Common newt	69.84	0.658	0.698	0.528	0.603	0.698	0.619
Great crested newt	88.36	0.790	0.884	0.539	0.818	0.884	0.834
Average	73.24	0.682	0.732	0.589	0.666	0.732	0.684

**Table 15:** Performance metrics for Anuran-Calls-(MFCCs) classification in FMLL.

Anuran-Calls-(MFCCs)	Accuracy	Precision	TNR	ROC	PRC	Recall	F-Score
Family	95.75	0.957	0.980	0.978	0.964	0.957	0.957
Genus	94.19	0.941	0.991	0.979	0.943	0.942	0.941
Species	93.55	0.935	0.992	0.983	0.935	0.936	0.935
Average	94.50	0.944	0.988	0.980	0.947	0.945	0.944

**Table 16:** Performance metrics for categories of HackerEarth-Adopt-A-Buddy dataset in FMLL.

HackerEarth-Adopt-A-Buddy	Accuracy	Precision	TNR	ROC	PRC	Recall	F-Score
Breed_category	85.43	0.856	0.927	0.965	0.938	0.854	0.850
Pet_category	86.80	0.869	0.928	0.946	0.928	0.868	0.865
Average	86.12	0.863	0.928	0.956	0.933	0.861	0.858

**Table 17:** The comparison of FMLL with state-of-the-art methods using the Amphibians dataset.

Method	Accuracy
Gradient-Boosted Trees (GBT) [124]	64.18
Random Forest (RF) [124]	57.54
AdaBoost (ADA) [124]	60.01
Decision Tree (DT) [124]	58.37
Partially Monotonic Decision Tree (PMDT) [128]	71.50
Average	62.32
Proposed (FMLL with BR and REPTree)	73.24

**Table 18:** The comparison of FMLL with state-of-the-art methods [125] using the Anuran-Calls-(MFCCs) dataset.

Method	Precision	Recall	F-Score
Species			
KNN-Flat	0.690	0.720	0.700
RBF-SVM-Flat	0.850	0.540	0.660
Polynomial-SVM-Flat	0.710	0.760	0.740
Tree-Flat	0.490	0.500	0.500
KNN-LCPL	0.691	0.719	0.705
KNN-Hierarchical-LCPN	0.690	0.720	0.700
RBF-SVM- Hierarchical-LCPN	0.840	0.540	0.650
Polynomial-SVM-Hierarchical-LCPN	0.680	0.710	0.700
Tree-Hierarchical-LCPN	0.570	0.560	0.560
KNN-Hierarchical-LCPL	0.690	0.720	0.700
RBF-SVM- Hierarchical-LCPL	0.830	0.520	0.640
Polynomial-SVM-Hierarchical-LCPL	0.690	0.740	0.720
Tree-Hierarchical-LCPL	0.470	0.500	0.490
Proposed (FMLL with BR and REPTree)	0.935	0.936	0.935
Family			
KNN-LCPL	0.713	0.820	0.763
Proposed (FMLL with BR and REPTree)	0.957	0.957	0.957
Genus			
KNN-LCPL	0.663	0.731	0.695
Proposed (FMLL with BR and REPTree)	0.941	0.942	0.941
Species + Family + Genus			
KNN-LCPL	0.689	0.757	0.721
Proposed (FMLL with BR and REPTree)	0.944	0.945	0.944

## Data Availability

The “Amphibians” dataset [124] is publicly available in the UCI (University of California Irvine) machine learning repository (https://archive.ics.uci.edu/dataset/528/amphibians, accessed on 22 April 2024). The “Anuran-Calls-(MFCCs)” dataset [125] is publicly available in the UCI learning repository (https://archive.ics.uci.edu/dataset/406/anuran+calls+mfccs, accessed on 22 April 2024). The “HackerEarth-Adopt-A-Buddy” dataset [126] is publicly available in the Kaggle machine learning repository (https://www.kaggle.com/datasets/mannsingh/hackerearth-ml-challenge-pet-adoption, accessed on 22 April 2024).

## Abbreviations

The following abbreviations are used in this paper.

AI	Artificial intelligence
ANN	Artificial neural network
ADA	AdaBoost
AMFSC	Amendable multi-function sensor control method
AR	Additive regression
BR	Binary relevance
CC	Classifier chains
CNN	Convolutional neural network
CPPS	Cyber-physical production system
DAGs	Dual attention gates
DeepFedWT	Federated deep learning framework
DPLA	Differential privacy Laplace mechanism
DT	Decision tree
ECC	Ensemble of classifier chains
EIAs	Environmental impact assessments
ETo	Estimation of the evapotranspiration
FCLOpt	Federated contrastive learning optimization
FedAAR	Federated learning framework for animal activity recognition
FedAvg	Federated averaging
FELIDS	Federated learning-based intrusion detection system
FL	Federated learning
FMLL	Federated multi-label learning
FPR	False positive rate
FTL	Federated transfer learning
GBT	Gradient-boosted tree
GIS	Geographic information system
GNN	Graph neural network
HFL	Horizontal federated learning
IDS	Intrusion detection system
IIoT	Industrial Internet of things
IoT	Internet of things
KNN	K-nearest neighbors
LCPL	Local classifier per level
LCPN	Local classifier per node
LP	Label powerset
LR	Logistic regression
LSM	Landslide susceptibility map
LSTM	Long short-term memory
MAE	Absolute error
MetaMIML	Meta learning based multi-instance multi-label learning
ML	Machine learning
MLC	Multi-label classification
ML-CookGAN	Multi-label generative adversarial network
ML-kNN	Multi-label k-nearest neighbors
MMVFL	Multi-participant multi-class vertical federated learning
MSRAN	Multi scale residual attention network
PC	Pairwise coupling
PMDT	Partially monotonic decision tree
PRC	Precision-recall curve
RAkEL	Random k-labelsets
RBF	Radial basis function
RC	Random committee
RD	Regression by discretization
REPTree	Reduced error pruning tree
RF	Random forests
RMSE	Root mean square error
RNN	Recurrent neural network
ROC	Receiver operating characteristic
RTF-REPTree	Rotational forest and reduced error pruning trees
SCADA	Supervisory control and data acquisition
SSA	Sparrow search algorithm
SVM	Support vector machines
TNR	True negative rate
TPR	True positive rate
VFL	Vertical federated learning
